# Extensively drug-resistant *Pseudomonas aeruginosa*: clinical features and treatment with ceftazidime/avibactam and ceftolozane/tazobactam in a tertiary care university hospital center in Portugal – A cross-sectional and retrospective observational study

**DOI:** 10.3389/fmicb.2024.1347521

**Published:** 2024-02-13

**Authors:** Diogo Mendes Pedro, Sérgio Eduardo Paulo, Carla Mimoso Santos, Ana Bruschy Fonseca, José Melo Cristino, Álvaro Ayres Pereira, Cátia Caneiras

**Affiliations:** ^1^Serviço de Doenças Infeciosas, Centro Hospitalar Universitário Lisboa Norte EPE, Lisbon, Portugal; ^2^Clínica Universitária de Doenças Infeciosas, Faculdade de Medicina da Universidade de Lisboa, Lisbon, Portugal; ^3^Instituto de Farmacologia e Neurociências, Faculdade de Medicina da Universidade de Lisboa, Lisbon, Portugal; ^4^Laboratório de Microbiologia na Saúde Ambiental, Laboratório Associado TERRA, Instituto de Saúde Ambiental, Faculdade de Medicina da Universidade de Lisboa, Lisbon, Portugal; ^5^Unidade Local do Programa de Prevenção e Controlo de Infeções e das Resistências aos Antimicrobianos, Centro Hospitalar Universitário Lisboa Norte EPE, Lisbon, Portugal; ^6^Serviço de Patologia Clínica, Centro Hospitalar Universitário Lisboa Norte EPE, Lisbon, Portugal; ^7^Instituto de Microbiologia, Faculdade de Medicina da Universidade de Lisboa, Lisbon, Portugal; ^8^Egas Moniz Center for Interdisciplinary Research (CiiEM), Egas Moniz School of Health and Science, Monte da Caparica, Portugal; ^9^Instituto de Medicina Preventiva e Saúde Pública, Faculdade de Medicina da Universidade de Lisboa, Lisbon, Portugal

**Keywords:** extensively drug-resistant, *Pseudomonas aeruginosa*, antimicrobial resistance, difficult-to-treat infections, ceftazidime-avibactam, ceftolozane-tazobactam

## Abstract

**Introduction:**

Extensively drug-resistant *Pseudomonas aeruginosa* (XDR-PA) is a growing concern due to its increasing incidence, limited therapeutic options, limited data on the optimal treatment, and high mortality rates. The study aimed to characterize the population, the outcome and the microbiological characteristics of XDR-PA identified in a Portuguese university hospital center.

**Methods:**

All XDR-PA isolates between January 2019 and December 2021 were identified. XDR-PA was defined as resistance to piperacillin-tazobactam, third and fourth generation cephalosporins, carbapenems, aminoglycosides and fluoroquinolones. A retrospective analysis of the medical records was performed.

**Results:**

One hundred seventy-eight individual episodes among 130 patients with XDR-PA detection were identified. The most common sources of infection were respiratory (32%) and urinary tracts (30%), although skin and soft tissue infections (18%) and primary bacteremia (14%) were also prevalent. Colonization was admitted in 64 cases. Several patients had risk factors for complicated infections, most notably immunosuppression, structural lung abnormalities, major surgery, hemodialysis or foreign intravascular or urinary devices. XDR-PA identification was more frequent in male patients with an average age of 64.3 ± 17.5 years. One non-susceptibility to colistin was reported. Only 12.4% were susceptible to aztreonam. Ceftazidime-avibactam (CZA) was susceptible in 71.5% of the tested isolates. Ceftolozane-tazobactam (C/T) was susceptible in 77.5% of the tested isolates. Antibiotic regimens with XDR-PA coverage were reserved for patients with declared infection, except to cystic fibrosis. The most frequently administered antibiotics were colistin (41 cases), CZA (39 cases), and C/T (16 cases). When combination therapy was used, CZA plus colistin was preferred. The global mortality rate among infected patients was 35.1%, significantly higher in those with hematologic malignancy (50.0%, *p* < 0.05), followed by the ones with bacteremia (44.4%, *p* < 0.05) and those medicated with colistin (39.0%, *p* < 0.05), especially the ones with respiratory infections (60.0%). Among patients treated with CZA or C/T, the mortality rate seemed to be lower.

**Discussion:**

XDR-PA infections can be severe and difficult to treat, with a high mortality rate. Even though colistin seems to be a viable option, it is likely less safe and efficient than CZA and C/T. To the best of the authors’ knowledge, this is the first description of the clinical infection characteristics and treatment of XDR-PA in Portugal.

## 1 Introduction

Extensively drug resistant *Pseudomonas aeruginosa* (XDR-PA) is a growing concern due to its increasing incidence, limited therapeutic options, limited data on the optimal treatment, and high mortality rates. Being able to survive in many ecological settings, *P. aeruginosa* is highly flexible and can adapt to a wide array of environmental pressures. Having access to numerous metabolic pathways and a wide range of virulence and resistance factors thanks to its genetic plasticity, make it one of the most successful bacteria in Medical Microbiology (Behzadi et al., 2022a,b).

Indeed, although in the late 20th century the incidence of *P. aeruginosa* bloodstream infections was declining, by 2010 it had increased again to 6.5 per 10,000 (Werth et al., 2015). Moreover, an increase in the prevalence of multidrug resistant (MDR) and XDR-PA was evident, with XDR-PA prevalences ranging from 2.6 to 11.2% being described (Walkty et al., 2017; Sader et al., 2018a). Furthermore, according to the European Center for Disease Prevention and Control (ECDC), in 2021 most countries in Europe reported rates of resistance higher than 10% for the majority of the antimicrobial classes under surveillance and only two countries reported less than 5% resistance to carbapenems (European Centre for Disease Prevention and Control and World Health Organization, 2023). In Portugal, in 2021, 12.7% of all isolates were resistant to at least three classes. The highest resistance was seen to fluoroquinolones (18.1%) and piperacillin-tazobactam (16.4%). The most susceptible of the five surveilled classes was aminoglycosides (6.3% resistant) (European Centre for Disease Prevention and Control and World Health Organization, 2023). Moreover, carbapenem-resistant *P. aeruginosa* is of critical priority in the World Health Organization Priority Pathogens List (Behzadi et al., 2022b).

Even though *P. aeruginosa* is often a common cause of severe bloodstream infection, ventilator-associated pneumonia and other hospital-acquired infections (Bassetti et al., 2018b), XDR-PA carries an increased mortality. A meta-analysis by *Matos et al.* describes a mortality of 44.6% among patients infected with MDR *P. aeruginosa* versus 24.8% in those with non-MDR infections (de Matos et al., 2018). Recio et al. (2018) compared patients with bacteremia due XDR-PA versus susceptible strains and also found a difference in mortality (62.5% versus 30%).

Even though it is an opportunistic pathogen present in the environment, *P. aeruginosa* is seldom found in the microbiota of healthy humans (Silby et al., 2011; Estepa et al., 2014). Nevertheless, in patients at risk, such as those with a large exposure to the healthcare setting or with certain chronic illnesses (like cystic fibrosis), the colonization rate can reach up to 80% (Gómez-Zorrilla et al., 2014; Ciofu et al., 2015), while those submitted to antibiotic therapy have a higher risk of attaining MDR strains (Gómez-Zorrilla et al., 2014). It also has intrinsic resistance to many antimicrobial drugs and a high capacity of attaining resistance mutations and mobile genetic elements (Bassetti et al., 2018a,b; Horcajada et al., 2019). Although there are many factors contributing to the emergence of antimicrobial resistance, the misuse or overuse of antibiotics is paramount (Algammal et al., 2023). Indeed, despite the new antimicrobials or β-lactam inhibitors that have become commercially available in the last 5 years (Karvouniaris et al., 2023), the emergence of antimicrobial resistance remains one of the greatest threats to global health (Mendes et al., 2022).

Despite the existence of several articles depicting the molecular epidemiology and resistance mechanisms of *P. aeruginosa*, to the best of the authors’ knowledge there is a gap in the clinical characterization of these patients in Portugal, a country with one of the highest antimicrobial resistance rates in Europe (Pereira et al., 2013, 2015; Hernández-García et al., 2021a,b,c). The authors aim to characterize the population, the outcome and the microbiological characteristics of XDR-PA identified in a tertiary care university hospital center in Portugal.

## 2 Materials and methods

### 2.1 Study procedures

**FIGURE 1:**
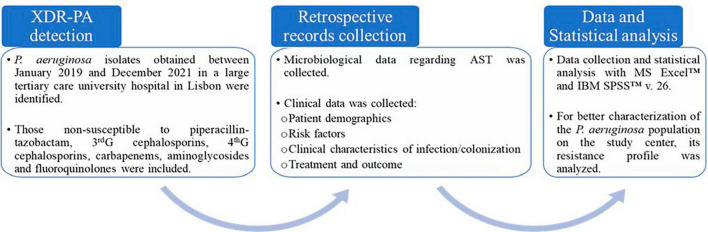
A flowchart depicting the procedures done in the present study.

A cross-sectional and retrospective observational study was performed in a 1,000-bed tertiary care university hospital center in Lisbon, including inpatients and outpatients with XDR-PA detection between January 2019 and December 2021 (Figure 1). XDR-PA was defined as non-susceptibility to piperacillin-tazobactam, third and fourth generation cephalosporins, carbapenems, aminoglycosides and fluoroquinolones, which is included in the consensus defined by Magiorakos et al. (2012).

Electronic medical records were obtained and analyzed. The following population characteristics were collected: gender and age, risk factors such as presence of immunosuppressive status, invasive devices and certain chronic illnesses, site of infection or colonization, antimicrobial therapy performed and outcome. Colonization was defined as the positive identification of XDR-PA without clinical evidence of infection.

Statistical analysis was performed with MS Excel™ and IBM SPSS™ version 26. A descriptive analysis of the variables was performed. When comparing categorical variables, the Pearson’s Chi-square test was used. Applicability conditions were verified. The significance level was set at *p* < 0.05.

### 2.2 Bacterial identification and antimicrobial susceptibility testing

Bacterial identification and antimicrobial susceptibility testing were routinely performed at the hospital’s microbiology laboratory by automated systems (MicroScan WalkAway™ or Vitek™).

Susceptibility was tested by a panel of antibiotics: piperacillin-tazobactam, ceftazidime, cefepime, imipenem, meropenem, amikacin, gentamycin, and ciprofloxacin. When there were limited treatment options, ceftazidime-avibactam, ceftolozane-tazobactam, aztreonam, fosfomycin and colistin were also tested. The colistin susceptibility determination was been performed by microdilution. The clinical breakpoints were interpreted in accordance with European Committee on Antimicrobial Susceptibility Testing (EUCAST) Clinical breakpoints - breakpoints and guidance, available at the European Society of Clinical Microbiology and Infectious Diseases (ESCMID) website.^1^ The isolates were then categorized as susceptible and resistant by applying the breakpoints results.

When the production of metallo-beta lactamases was suspected, a GeneXpert™ assay was performed.

### 2.3 XDR-PA resistotyping

To better characterize the XDR-PA population, we performed a resistotype distribution analysis. Considering that all included XDR-PA isolates were by definition non-susceptible to piperacillin-tazobactam, third- and fourth generation cephalosporins, carbapenems, aminoglycosides and fluoroquinolones, the resistance patterns of those with available susceptibility results to colistin, ceftazidime-avibactam, ceftolozane-tazobactam, aztreonam, and fosfomycin were analyzed. Resistance pattern distribution (resistotyping) and MAR index of clinical XDR *P. aeruginosa* isolates were performed in accordance with previous studies (Behzadi et al., 2022b).

## 3 Results

Over the 3 years considered in this study (2019-2021) a total of 6,514 isolates of *P. aeruginosa* pertaining to 3,181 patients were identified. Considering XDR-PA, a total of 243 isolates pertaining to 178 episodes concerning 130 patients were found. The prevalence of XDR-PA identification among all *P. aeruginosa* isolates was 3.7%, corresponding to 4.1% of all patients with identification of this microorganism. A new episode was defined as a positive XDR-PA isolate when obtained more than 14 days after the last one, in the same patient. Of these 178 episodes, 114 (64.0%) were from infections and 64 (36.0%) were from colonizations. Moreover, 69 episodes (48 patients) happened in 2019, 61 (46 patients) occurred in 2020 and 48 (36 patients) in 2021. It is noteworthy that only six patients were co-infected with SARS-CoV-2, of which four of them died.

### 3.1 Clinical results

Patient demographics of the 130 patients in which was recovered an XDR-PA isolate are described in Table 1. The cohort was predominantly male (65.4%), with a mean age of 64.3 ± 17.5 years. The most frequent comorbidities described in patients with XDR-PA were diabetes mellitus, immunosuppressive therapy, the presence of a urinary tract device, malignancy and hemodialysis or renal transplant.

**TABLE 1:** Patient demographics of the 130 patients with XDR *Pseudomonas aeruginosa* (XDR-PA) and site of infection/colonization of the 178 XDR-PA episodes.

Patients demographics *N* = 130 patients	
Age (years):	
Mean ± SD	64.3 ± 17.5
Median	65
Min. – Max.	13–98
**Gender:**	**Number of patients** ***n* (%)**
Male	85 (65.4%)
Female	45 (34.6%)
**Main comorbidities:**	***n* (%)**
Diabetes mellitus	29 (22.3%)
Immunosuppressive therapy	28 (21.5%)
Urinary tract device	18 (13.8%)
Hematologic malignancy	18 (13.8%)
Solid neoplasm	14 (10.8%)
Renal transplant	13 (10.0%)
Chronic kidney disease in hemodialysis	12 (9.2%)
Chronic skin lesions	10 (7.7%)
Cystic fibrosis	9 (6.9%)
Other chronic lung diseases	8 (6.2%)
Complicated intra-abdominal surgery	7 (5.4%)
Tracheostomy	5 (3.8%)
Hepatic cirrhosis	3 (2.3%)
HIV/AIDS	1 (0.8%)
Common variable immunodeficiency	1 (0.8%)
**Site of infection/colonization** ***N* = 178 episodes**	**Number of isolates** ***n* (%)**
**Infections**	**114 (64.0%)**
Respiratory	36 (31.6%)
Urinary tract	34 (29.8%)
Skin and soft tissues	20 (17.5%)
Primary bacteremia	16 (14.0%)
Intra-abdominal	6 (5.3%)
Ear	1 (0.9%)
Eye	1 (0.9%)
**Colonization – *N* (%)**	**64 (36.0%)**
Respiratory tract	24 (37.5%)
Urinary tract	22 (34.4%)
Skin and soft tissues	18 (28.1%)

Extensively drug-resistant *Pseudomonas aeruginosa* (XDR-PA) was the etiology of infection in several organs and systems. In Table 1 there is a description of the main sources of infection. A total 114 infections were found, most frequently in the respiratory tract, urinary tract and skin and soft tissues. Sixteen (14.0%) cases presented with primary bacteremia. Of these, most were in immunosuppressed patients often with hematological malignancy. Data regarding mechanical ventilation or the presence of a central venous access was unavailable. It is noteworthy that 23.7% (27/114) of all infections presented with bacteremia. In 64 episodes XDR-PA identification was considered colonization, most frequently of the respiratory and urinary tracts.

When infected, patients were submitted to either monotherapy (52 episodes) with an active drug according to the antimicrobial susceptibility test, or to combined therapy (32 episodes). Whenever a patient was prescribed an active drug combined with another to which this agent was resistant (i.e., colistin plus meropenem), it was considered monotherapy. In 30 cases the information regarding appropriate antibiotic therapy was unavailable.

Table 2 describes the appropriate antimicrobials and combinations used and properly documented. Colistin was used in 41 episodes, ceftazidime-avibactam in 39 and ceftolozane-tazobactam in 16. Aztreonam was used in seven cases, but only once in monotherapy. Fosfomycin was used in seven cases but only in combination.

**TABLE 2:** Antimicrobial treatment to *Pseudomonas aeruginosa* infections and all-cause mortality by treatment.

Monotherapy ± inhaled therapy	Number of patients	All-cause mortality *n* (%)
Colistin	21	6 (28.6%)
Ceftazidime-avibactam	19	3 (15.8%)
plus inhaled colistin	3	0 (0.0%)
Ceftolozane-tazobactam	13	2 (15.4%)
Aztreonam	1	0 (0.0%)
**Combination therapy ± inhaled therapy**		
Ceftazidime-avibactam plus colistin	12	5 (41.7%)
plus inhaled colistin	1	0 (0.0%)
Ceftazidime-avibactam plus aztreonam	4	3 (75.0%)
Ceftazidime-avibactam plus fosfomycin	4	0 (0.0%)
Ceftolozane-tazobactam plus colistin	3	1 (33.3%)
Colistin plus aztreonam	2	1 (50.0%)
plus inhaled colistin	1	0 (0.0%)
Colistin plus fosfomycin	3	3 (100.0%)
**Exclusively inhaled therapy^1^**		
Inhaled colistin	2	0 (0.0%)
Inhaled aztreonam	1	0 (0.0%)
Inhaled colistin plus inhaled aztreonam	2	0 (0.0%)
Inhaled colistin plus inhaled tobramycin	1	0 (0.0%)

^1^In patients with cystic fibrosis.

Colonized patients were not submitted to antimicrobial therapy directed to the XDR-PA except in six episodes of cystic fibrosis that presented with respiratory colonization and who were under inhaled colistin (two cases), inhaled aztreonam (one case), both in combination (two cases) or inhaled colistin plus inhaled tobramycin (one case).

There were 40 deaths (35.1%) among infected patients (Table 3). The ones who were treated with combined therapy had a higher mortality (43.8%) than those that were treated with monotherapy (21.2%, *p* > 0.05). Adjusting for the antimicrobial used, the mortality rate was 39.0% (*p* > 0.05, 16 deaths) among colistin-treated patients, 28.2% (11 deaths) among ceftazidime-avibactam-treated patients and 18.8% (three deaths) among ceftolozane-tazobactam-treated patients. Considering only patients with respiratory disease, colistin therapy was associated with a mortality rate of 60.0% as opposed to 32.3% when excluding these patients. Comparison of mortality between patients treated with different antibiotics failed to yield statistically significant differences.

**TABLE 3:** Clinical outcome of infection considering source of infection and treatment including colistin, ceftazidime-avibactam or ceftolozane-tazobactam.

	All cause mortality in infected patients deaths/number of patients (%)	
**Source of infection**		
All sources of infection	40/114 (35.1%)	*p > 0.05*
Respiratory	17/36 (47.2%)	*p > 0.05*
Urinary tract	9/34 (26.5%)	*p > 0.05*
Skin and soft tissues	7/20 (35.0%)	*p > 0.05*
Primary bacteremia	4/16 (25.0%)	*p > 0.05*
Intra-abdominal	2/6 (33.3%)	*p > 0.05*
Ear	1/1 (100.0%)	*p > 0.05*
Eye	0/1 (0.0%)	*p > 0.05*
Any source, with bacteremia	12/27 (44.4%)	***p* < *0.05***
**Treatment**		
Among colistin-treated patients	16/41 (39.0%)	***p* < *0.05***
Respiratory source	6/10 (60.0%)	*p > 0.05*
Non-respiratory source	10/31 (32.3%)	*p > 0.05*
Among CZA-treated patients	11/39 (28.2%)	*p > 0.05*
Among C/T-treated patients	3/16 (18.8%)	*p > 0.05*
Monotherapy vs. Combined therapy	11/52 (21.2%) *Vs.* 14/32 (43.8%)	***p* < 0.05**

CZA, ceftazidime-avibactam; C/T, ceftolozane-tazobactam. The bold value shows when *p*-value was less than 0.05.

**FIGURE 2:**
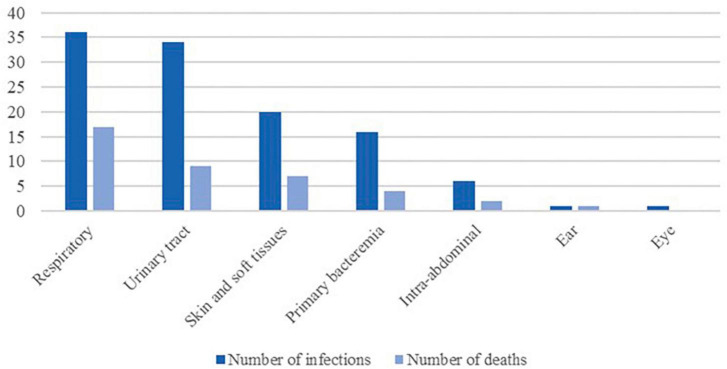
Frequency of infections and all causes mortality by source of infection.

Regarding the source of infection (Figure 2), mortality was highest among patients with respiratory infections (47.2%), followed by those with skin and soft tissues infections (35.0%), intra-abdominal infections (33.3%), and urinary tract infections (26.5%). Among patients with positive blood cultures the mortality rate was 44.4% (*p* < 0.05), although only 25.0% when presenting with primary bacteremia. Considering all comorbidities, only hematologic malignancy was significantly associated with mortality (50.0% died, *p* < 0.05).

### 3.2 Microbiological results

Extensively drug-resistant *Pseudomonas aeruginosa* (XDR-PA) was identified in 243 microbiological examinations, corresponding to the 178 episodes. Antimicrobial susceptibility testing (AST) was performed in 216 (88.9%) isolates (Table 4). In the remaining, AST was not performed since it had already been done for another contemporary sample.

**TABLE 4:** Antimicrobial susceptibilities of XDR-PA isolates to selected antimicrobial agents.

*N* (%)	Colistin	Ceftazidime-avibactam	Ceftolozane-tazobactam	Aztreonam	Fosfomycin
Tested isolates^1^	216 (88.9%)	158 (65.0%)	173 (71.2%)	188 (77.4%)	98 (40.3%)
Susceptible (S)^2^	215 (99.5%)	113 (71.5%)	134 (77.5%)	26 (13.8%)	22 (22.4%)
Non-susceptible (NS)^2^	1 (0.5%)	45 (28.5%)	39 (22.5%)	162 (86.2%)	76 (77.6%)

All isolates were resistant to piperacillin-tazobactam, third and fourth generation cephalosporins, carbapenems, aminoglycosides and fluoroquinolones.

^1^Percentage in relation to all isolates.

^2^Percentage in relation to the tested isolates.

The only resistance found to colistin was in a 34-year-old patient with cystic fibrosis, and it remained susceptible exclusively to ceftazidime-avibactam and to ceftolozane-tazobactam novel combinations. Moreover, a 56-year-old patient with hematologic malignancy under chemotherapy presented with febrile neutropenia and primary bacteremia with a VIM-producing XDR-PA that was susceptible only to colistin. The patient was treated with colistin and fosfomycin and ultimately died.

**FIGURE 3:**
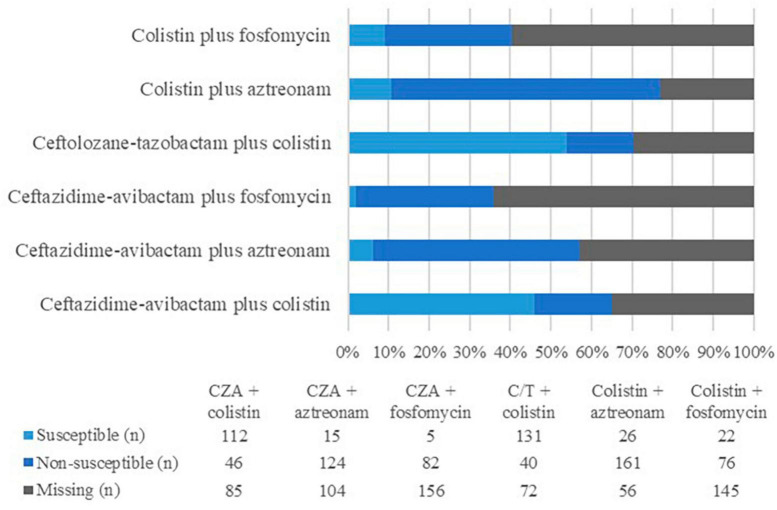
Antimicrobial susceptibilities of XDR-PA isolates (*n* = 243) to antimicrobial combinations used as treatment in the present study. CZA, ceftazidime-avibactam; C/T, ceftolozane-tazobactam.

Among all *P. aeruginosa* isolates, the yearly susceptibility rate to meropenem varied between 80 and 82%. Regarding XDR-PA isolates, when tested, most were susceptible to ceftazidime-avibactam and ceftolozane-tazobactam, while only 22.4% were susceptible to fosfomycin and 12.4% to aztreonam. Regarding the strains tested both for susceptibility to ceftazidime-avibactam and to ceftolozane-tazobactam, 63.2% were susceptible and 19.1% were resistant to both antibiotics. Of those resistant to ceftazidime-avibactam, 42.2% were susceptible to ceftolozane-tazobactam, while of those resistant to ceftolozane-tazobactam, 16.1% were susceptible to ceftazidime-avibactam. The related defaults for XDR in association with the used antibiotics and combinations are presented in Figure 3. Most of the strains tested for both colistin and ceftazidime-avibactam and colistin and ceftolozane-tazobactam were susceptible to both antimicrobials (70.9 and 76.6%, respectively). Two-antibiotic combinations with fosfomycin or aztreonam were less likely to be susceptible to both drugs, which is in accordance with the lower susceptibility rates found in those antimicrobials.

### 3.3 XDR-PA resistotyping

Table 5 shows the resistance patterns of 65 clinical XDR-PA that were non-susceptible to piperacillin-tazobactam, third and fourth generation cephalosporins, carbapenems, aminoglycosides and fluoroquinolones and that, additionally had available AST results for colistin, ceftazidime-avibactam, ceftolozane-tazobactam, aztreonam and fosfomycin. Twelve different resistotypes were found.

**TABLE 5:** Resistance patterns distribution and the MAR indices of clinical XDR-PA.

Resistotype	Resistance pattern	MAR index	Ratio *n* (%)
I	Ceftolozane-tazobactam	0,2	1 (1.5%)
II	Aztreonam	0,2	3 (4.6%)
III	Fosfomycin	0,2	2 (3.1%)
IV	Ceftazidime-avibactam, ceftolozane-tazobactam	0,4	3 (4.6%)
V	Ceftazidime-avibactam, aztreonam	0,4	1 (1.5%)
VI	Ceftazidime-avibactam, fosfomycin	0,4	1 (1.5%)
VII	Aztreonam, fosfomycin	0,4	26 (40.0%)
VIII	Ceftazidime-avibactam, ceftolozane-tazobactam, aztreonam	0,6	8 (12.3%)
IX	Ceftazidime-avibactam, ceftolozane-tazobactam, fosfomycin	0,6	1 (1.5%)
X	Ceftazidime-avibactam, aztreonam, fosfomycin	0,6	13 (20.0%)
XI	Ceftolozane-tazobactam, aztreonam, fosfomycin	0,6	2 (3.1%)
XII	Ceftazidime-avibactam, ceftolozane-tazobactam, aztreonam, fosfomycin	0,8	4 (6.2%)
**Total**			**65 (100.0%)**

All isolates were resistant to piperacillin-tazobactam, third and fourth generation cephalosporins, carbapenems, aminoglycosides and fluoroquinolones.

The most common resistotype was the combined resistance to aztreonam and fosfomycin (*n* = 26, 40.0%), followed by ceftazidime-avibactam, aztreonam and fosfomycin (*n* = 13; 20.0%), and ceftazidime-avibactam, ceftolozane-tazobactam and aztreonam (*n* = 8; 12.3%). Resistance to at least two of the tested antibiotics occurred in 90.8%, while 43.1% were resistant to more than three antimicrobials.

## 4 Discussion

Extensively drug resistant *Pseudomonas aeruginosa* (XDR-PA) is a growing concern due to its increasing incidence, limited therapeutic options, limited data on the optimal treatment, and high mortality rates. Even though the criteria used to define XDR-PA did not match exactly those defined by Magiorakos et al. (2012), the prevalence reported in the present study is lower than the one displayed in 2017 by a large-scale Spanish multicenter study (17%) (Del Barrio-Tofinõ et al., 2019). Similarly, a trial including patients with ventilator-associated pneumonia in Spain, Greece and Italy showed a rate of 35.8% XDR isolates, mostly from Greece (Pérez et al., 2019). Other countries around the world also reported higher XDR-PA prevalence, such as 22.1% out of 447 *P. aeruginosa* isolates in Nepal (Mahto et al., 2021) or 15.5% out of 3248 isolates in Iran (Mirzaei et al., 2020). The prevalence reported in the present study is more similar to that described in Canada and in USA. McCracken et al. (2019) showed 4.5% XDR-PA among all 3864 Canadian isolates between 2007 and 2016, while Sader et al. (2017) depict 9.4% XDR-PA among 7452 American isolates from 2012 to 2015. Although there is a lack in data regarding the prevalence of XDR-PA in Portuguese isolates, ECDC reports a resistance rate to carbapenems of 14.1 and of 12.7% to any combination of at least three of the following antibiotics: piperacillin-tazobactam, ceftazidime, carbapenems, aminoglycosides and fluoroquinolones (Antimicrobial resistance surveillance in Europe 2023 - 2021 data. Stockholm: European Centre for Disease Prevention and Control and World Health Organization, 2023).

While *P. aeruginosa* is rarely found in the microbiota of healthy humans (Silby et al., 2011; Estepa et al., 2014), it can colonize up to 80% of patients with risk factors, such as a large exposure to the healthcare setting or certain chronic illnesses (such as cystic fibrosis, and solid or hematologic malignancies) (Gómez-Zorrilla et al., 2014; Ciofu et al., 2015). The presence of foreign devices, such as venous or urinary catheters, tracheostomy (especially in children), open abdominal surgery, diabetes, chronic hepatic disorder and end-stage renal disease also increase the risk for *P. aeruginosa* infection (Varaiya et al., 2008; Willmann et al., 2014; Miller et al., 2016; Bassetti et al., 2018a; Russell et al., 2019; Jean et al., 2020; Körpinar, 2021). Also prior antibiotic therapy (especially fluoroquinolones and carbapenems), so frequent in the healthcare setting and in patients with these comorbidities, is a major risk factor for attaining MDR strains (Falagas et al., 2006; Peña et al., 2012; Gómez-Zorrilla et al., 2014; Bassetti et al., 2018a; Jean et al., 2020). In fact, prior use of fluoroquinolones or carbapenems has been depicted as an independent risk factor for XDR-PA infections (Palavutitotai et al., 2018).

In *P. aeruginosa*, resistance mechanisms such as decreased permeability, expression of efflux pumps, target modifications and production of inactivating enzymes have all been described (Mesaros et al., 2007). In particular, production of extended-spectrum beta-lactamases and carbapenemases have been described, including metallo-beta-lactamases (Mesaros et al., 2007; Sacha et al., 2008; Ríos et al., 2018; Muddassir et al., 2021). Since these enzymes use zinc, which seems to be a valuable cofactor to hydrolyze beta-lactams, instead of serine in their active sites, they confer resistance to all beta-lactam antibiotics except aztreonam, while not being degraded by currently available beta-lactamase inhibitors (Sacha et al., 2008; Behzadi et al., 2020). In this study there was one VIM-producing XDR-PA that was susceptible only to colistin. Despite VIM not being able to degrade aztreonam, *P. aeruginosa* is capable of producing simultaneously additional inactivating enzymes, conferring resistance also to this drug (Mesaros et al., 2007; Ríos et al., 2018). So, the combination of aztreonam-avibactam (or ceftazidime-avibactam plus aztreonam if the former is not available) is pertinent as it combines the ability of avibactam to inactivate all serine-beta-lactamases, thus allowing aztreonam to be effective (Marshall et al., 2017; Mischnik et al., 2017). However, due to mechanisms of resistance to aztreonam independent of beta-lactamases, the activity of this combination has to be confirmed with synergy testing (Karakonstantis et al., 2020).

One drug that is often protected by the cross-resistance of other anti-pseudomonal antibiotics is colistin (Mesaros et al., 2007). And while pan-drug resistant isolates have been described, most often XDR-PA remains susceptible to colistin (Souli et al., 2008; Viedma et al., 2009; Giani et al., 2018). In this study all isolates but one were susceptible to colistin. However, while the prevalence of resistance to ceftazidime-avibactam and to ceftolozane-tazobactam appears to be overall low in Europe and the United States, the authors report approximately one quarter of non-susceptible isolates. This might be explained by the geographical variation of the predominance of different mechanisms of resistance (Flamm et al., 2014; Del Barrio-Tofiño et al., 2017; Giani et al., 2018; Livermore et al., 2018; Sader et al., 2018b; Evans et al., 2019), although when considering the cross-resistance of ceftolozane-tazobactam and ceftazidime-avibactam in this study, the authors believe the mechanism not to be predominantly related to carbapenemases. Regarding aztreonam and fosfomycin, the authors report high rates of non-susceptibility, as displayed by other centers and other Portuguese data (Sader et al., 2017; Safaei et al., 2017; Evans et al., 2019; Hernández-García et al., 2021a).

Regarding the analysis of the resistance patterns in XDR-PA isolates, it is not unexpected that the most frequent resistotype is resistance to both aztreonam and fosfomycin. It is noteworthy, however, that resistance patterns that included non-susceptibility to both aztreonam and ceftazidime-avibactam were frequent (40.0%, resistotypes V, VIII, X, and XII). Unfortunately, despite the knowledge that ceftazidime-avibactam resistance is emerging in *K. pneumoniae* in Portugal since its approval by the national regulatory authority in 2019 (Mendes et al., 2022, 2023), no data was available on synergy testing of ceftazidime-avibactam plus aztreonam or further study of resistance mechanisms in *P. aeruginosa*.

As XDR-PA is undoubtedly a growing threat with increasingly limited therapeutic options, it is essential to define new and innovative therapeutic strategies, either by developing new systemic drugs, new drug combinations, different methods of drug administration or alternative therapies (Mesaros et al., 2007; Marshall et al., 2017; Mischnik et al., 2017; Bassetti et al., 2018b; Horcajada et al., 2019; Ekkelenkamp et al., 2020; Gaurav et al., 2020; Yao et al., 2021). For example, the synergistic combination colistin-mefloquine has been suggested as a potential future therapeutic option against colistin-resistant strains due to its anti-biofilm activity (Behzadi et al., 2022a). Inhaled therapy has long since been used in cystic fibrosis and it can be a powerful adjunct in the treatment of XDR-PA infections. Since drug concentration in the lung is much higher with inhaled therapy than with the intravenous route, it can be effective even against *in vitro* resistant strains (Horcajada et al., 2019).

The authors report high mortality rates (35.1%), as observed in previous studies (Samonis et al., 2014; de Matos et al., 2018; Palavutitotai et al., 2018). Patients with hematologic malignancy have been described as being at an increased risk of a poor outcome (Samonis et al., 2014; Tofas et al., 2017; de Matos et al., 2018), perhaps due to their immunosuppressed status with frequent and recurrent infections and antimicrobial use. The authors report a 50.0% (*p* < 0.05) mortality among patients with hematologic malignancy.

Patients with bacteremia had a worse prognosis (mortality 44.4%, *p* < 0.05), similar to the 18.0% reported by Dantas et al. (2014) in their comparison between bacteremia due to susceptible and MDR strains. Having been established as an independent risk factor for mortality (Dantas et al., 2014), it surely is a marker for severe disease. Thus, it is interesting to note that in patients presenting with primary bacteremia the mortality was only 25.0%.

*Pseudomonas aeruginosa* is also a frequent cause of healthcare-associated respiratory and urinary tract infections (Quartin et al., 2013; Lamas Ferreiro et al., 2017; Jean et al., 2020), the most frequent sources of infection as reported in this study and consistent with the most frequent types of Healthcare Associated Infections, as reported by the ECDC (Suetens et al., 2023). Having been established that infections with resistant strains have a greater risk than susceptible strains (de Matos et al., 2018; Recio et al., 2018; Jean et al., 2020) it is not unexpected to find high mortality rates in this study. Nevertheless, 47.2% fatalities among patients with respiratory infections was higher than what had been previously described (Peña et al., 2013; Giaccari et al., 2021). The 26.5% mortality described among patients with urinary tract infections is similar to that described by Lamas Ferreiro et al. (2017).

Adjusting for the antimicrobial used, the authors report a higher mortality rate among colistin-treated patients (39.0%, *p* < 0.05). Although these results did not achieve statistical significance, it appears that the mortality among patients treated with ceftazidime-avibactam (28.2%) or ceftolozane-tazobactam (18.8%) was lower. Considering exclusively monotherapy, these results are consistent, with 28.6% of patients treated with colistin dying, against 15.8 and 15.4% of patients treated with ceftazidime-avibactam and ceftolozane-tazobactam, respectively. This seems paradoxical since colistin has been described as one of the most active antipseudomonal drugs (Del Barrio-Tofinõ et al., 2019; López Montesinos et al., 2021; Pinilla-Rello et al., 2021). The authors believe that this result might be related to both a better efficacy and safety profile of beta-lactams and a poorer penetration of colistin in the lung, which is supported by the 60.0% mortality when considering only patients with respiratory infection treated with colistin *versus* 32.3% when considering only other sources of infection. This findings support the last IDSA Guidance, where beta-lactams are put at the forefront of the treatment of MDR *P. aeruginosa* infections (Tamma et al., 2022). Patients that were submitted to combined directed therapy seemed to have a higher mortality rate than those treated with only one active drug. These results may be related to a possibly increased severity of disease among patients submitted to combined therapy.

The present study has several limitations. First, by using a narrower definition of XDR-PA than previously published, it is possible that other isolates were not considered. Therefore, comparisons with other studies must take this limitation into consideration. Second, several important variables, such as disease severity, exposure to venous catheterization or mechanical ventilation, recent or recurrent history of antibiotic therapy or healthcare exposure, and prior *P. aeruginosa* colonization status were most often not possible to ascertain.

The results of this study are relevant as they provide a first insight into the clinical outcome of Portuguese patients infected with XDR-PA, allowing for better care between relevant centers. However, in the future, it is essential to improve our understanding and to further characterize these patients, particularly in high-risk environments (such as focusing on Intensive Care Units and Hematology wards). Moreover, there is a crucial need for a current description of the molecular epidemiology and characterization of the resistance mechanisms of *P. aeruginosa* in Portugal. Furthermore, the integration of clinical data with molecular surveillance and the analysis of genetic resistance determinants may be relevant to improve the quality of care, allowing for a greater accent on research-based recommendations. Finally, with the potential impact of the COVID-19 pandemic on hospital epidemiology, it is paramount to evaluate this issue in the light of new therapeutic options.

## 5 Conclusion

In conclusion, patients infected with extensively drug resistant *Pseudomonas aeruginosa* are difficult to treat, with very limited therapeutic options. XDR-PA is a pathogen responsible for potentially severe disease that can have high mortality. Although the most frequent sources of infection were the respiratory and urinary sites, patients with bacteremia or hematologic malignancy had a higher risk. In this study, colistin was the most susceptible antibiotic *in vitro*. However, patients treated with ceftazidime-avibactam or ceftolozane-tazobactam appeared to have a better prognosis than those treated with colistin, especially considering those with respiratory infection. To the best of the authors’ knowledge, this is the first study to provide a clinical characterization of patients infected with XDR-PA strains in Portugal.

## Data availability statement

The raw data supporting the conclusions of this article will be made available by the authors, without undue reservation.

## Ethics statement

Ethical approval was not required for the study involving humans in accordance with the local legislation and institutional requirements. Written informed consent to participate in this study was not required from the participants or the participants’ legal guardians/next of kin in accordance with the national legislation and the institutional requirements.

## Author contributions

DM: Conceptualization, Formal analysis, Methodology, Writing – original draft. SP: Conceptualization, Methodology, Writing – review & editing. CS: Writing – review & editing. AF: Writing – review & editing. JM: Writing – review & editing. ÁP: Writing – review & editing. CC: Supervision, Writing – review & editing.

## Footnotes

1
https://www.eucast.org/clinical_breakpoints
